# Common gene signatures and molecular mechanisms of diabetic nephropathy and metabolic syndrome

**DOI:** 10.3389/fpubh.2023.1150122

**Published:** 2023-03-30

**Authors:** Chengyu Zhang, Han Li, Shixiang Wang

**Affiliations:** Department of Nephrology, Beijing Chao-Yang Hospital, Capital Medical University, Beijing, China

**Keywords:** transcription data, single-cell analysis, type 2 diabetes, oxidative phosphorylation, diabetic nephropathy

## Abstract

**Background:**

Diabetic nephropathy (DN) is the leading cause of end-stage renal disease. Multiple metabolic toxicities, redox stress, and endothelial dysfunction contribute to the development of diabetic glomerulosclerosis and DN. Metabolic syndrome (MetS) is a pathological state in which the body’s ability to process carbohydrates, fats, and proteins is compromised because of metabolic disorders, resulting in redox stress and renal remodeling. However, a causal relationship between MetS and DN has not been proven. This study aimed to provide valuable information for the clinical diagnosis and treatment of MetS with DN.

**Methods:**

Here, transcriptome data of DN and MetS patients were obtained from the Gene Expression Omnibus database, and seven potential biomarkers were screened using bioinformatics analysis. In addition, the relationship between these marker genes and metabolism and immune infiltration was explored. Among the identified marker genes, the relationship between *PLEKHA1* and the cellular process, oxidative phosphorylation (OXPHOS), in DN was further investigated through single-cell analysis.

**Results:**

We found that *PLEKHA1* may represent an important biomarker that perhaps initiates DN by activating B cells, proximal tubular cells, distal tubular cells, macrophages, and endothelial cells, thereby inducing OXPHOS in renal monocytes.

**Conclusion:**

Overall, our findings can aid in further investigation of the effects of drug treatment on single cells of patients with diabetes to validate PLEKHA1 as a therapeutic target and to inform the development of targeted therapies.

## 1. Introduction

Metabolic syndrome (MetS) is a group of medical conditions, including abdominal obesity, high blood pressure, high blood sugar, high triglyceride levels, and low high-density lipoprotein cholesterol levels, that increases the risk of developing cardiovascular disease and type 2 diabetes (1). MetS, independent of other covariates, is a predictor of declining renal function and worsening of albuminuria in patients with type 2 diabetes (2). Nephropathy remains a major cause of morbidity and a key determinant of mortality in patients with type 1 or type 2 diabetes mellitus (3, 4). Mitochondrial fatty acid β-oxidation is the preferred process for generating adenosine triphosphate (ATP) in the kidney, and its dysfunction results in ATP depletion and lipotoxicity, leading to tubular injury, inflammation, and subsequent progression of fibrosis (5).

The relationship between MetS and diabetic nephropathy (DN) is complex and bidirectional; while DN is considered a common progressive disease, MetS can be inhibited and it contributes to DN development and progression. The possible mechanisms of renal injury include insulin resistance, oxidative stress, increased production of pro-inflammatory cytokines, increased production of connective tissue growth and fibrotic factors, increased microvascular injury, and renal ischemia. MetS promotes kidney injury. However, despite the strong association between MetS and DN, a causal relationship has not yet been proven. Therefore, there is an urgent need to discover a range of novel biological markers for DN and MetS.

This study aimed to provide valuable information for the clinical diagnosis and treatment of MetS with DN. To this end, we analyzed transcriptomic RNA data from patients with DN and MetS and single-cell RNA sequencing (scRNA-seq) data from patients with DN to study their gene expression profiles and subsequently explore common biological markers of DN and MetS.

## 2. Materials and methods

### 2.1. Data sources

The keywords “diabetic nephropathy” and “metabolic syndrome” were used to search for DN and MetS gene expression profiles, respectfully, in the Gene Expression Omnibus (GEO) database (Home – GEO – NCBI [nih.gov]). Furthermore, the keywords “diabetic nephropathy” and “scRNA” were used to search for the single-cell dataset of DN. Finally, the GEO datasets GSE30529, GSE99340, and GSE98895 were selected. Four GEO datasets were selected —GSE30529, GSE98895, GSE99340, and GSE131882 (6–9). Information regarding the four datasets, including GSE numbers, detection platforms, samples, and types of RNA sources, is summarized in Supplementary material 1. GSE30529 and GSE98895 were also paired as a discovery cohort for weighted gene co-expression network analysis (WGCNA) and GSE99340 and GSE131882 as validated datasets for differential gene expression analysis. The gene expression profiles were then transformed, and the probes were matched to their gene symbols according to the annotation documents of the corresponding platforms. Finally, gene matrices with row names designating sample names and column names designating gene symbols were obtained for subsequent analysis.

The DN scRNA-seq dataset GSE131882 was downloaded from the GEO database, which included data for three control groups and three patients with DN (10). The original dataset contains data for 31,286 cells. The percentages of mitochondria and rRNA were then calculated using the PercentageFeatureSet function, with the genes expressed being >200 and <2,500, respectively. The selection criteria are shown in Supplementary Figure S1, while the workflow is shown in Supplementary Figure S2.

### 2.2. Identification of differentially expressed genes

Data normalization and probe annotation were performed on datasets GSE30529 and GSE98895 using the limma and GEOquery packages of R software version 4.1.3, with adjusted *p* < 0.05 and |log FC| > 1 as the DGE screening criteria (11). Subsequently, common DEGs for DN and MetS were obtained, and network enrichment analysis was performed using Metascape.^1^

### 2.3. Co-expression modules in DN and MetS

WGCNA is an algorithm that identifies co-expressed gene modules with high biological significance and explores the relationship between gene networks and diseases. WGCNA was used here to obtain DN- and MetS-associated modules. A total of 111,107 genes in the GEO dataset were obtained from the sequencing data and were used for WGCNA. The WGCNA package in R version 4.1.3 was used to perform the analysis. The appropriate soft-threshold powers (β, ranging from 1 to 20) were then selected using the “pickSoftThreshold” function in the WGCNA package based on the standard scale-free network. The soft-threshold power value β and gene correlation matrix among all gene pairs calculated by Pearson analysis were used to build an adjacency matrix. The topological overlap matrix and the corresponding dissimilarity were then transformed from the adjacency matrix. Subsequently, a hierarchical clustering dendrogram was built, with similar gene expression profiles divided into different modules. Finally, the expression profiles of each module were summarized by the module eigengene, and the correlation between the module eigengene and clinical features was calculated (12). Finally, modules with high correlation coefficients were targeted in terms of clinical features, and the genes in these modules were selected for subsequent analysis.

### 2.4. Identification of shared and unique gene signatures in DN and MetS

Least absolute shrinkage and selection operator (LASSO) was applied to identify DEGs with independent pre-diagnostic values. Based on the highest lambda value selected by 1,000 cross-validations in LASSO, a set of diagnostic genes and their LASSO coefficients were defined (13).

### 2.5. Construction of the XGBoost model

XGBoost was used to select a few key genomic features to build prediction models. XGBoost comprises an ensemble of K regression trees [T1(X, Y)…Tk(X, Y)], where X is the feature vector and Y is the corresponding risk. Assuming that the dataset contains *n* examples and *p* features D = {(xi, yi)} (|D| = *n*, xi ∈ X, yi ∈ Y), the ensemble XGBoost model uses K trees to predict patient risk:


$$\hat{y}i=\emptyset \left(x_{i}\right)=\overset{K}{\underset{K=1}{\sum }}\underset{k}{\int }\left(x_{i}\right),fk\in f$$

where *f* represents the space of the regression tree, *q* is the structure of the tree, T is the number of leaves in each tree, and $f_{k}$ represents the regression tree’s structure q with weight w. This method was implemented using the XGBoost package in R. All other parameters in our study used the default values in the R package XGBoost (14). The genes selected by XGBoost were considered candidate genes related to DN and MetS.

### 2.6. Analysis of immune cell infiltration and metabolic pathway

The CIBERSORT (https://cibersortx.stanford.edu/) deconvolution algorithm was used to evaluate differential immune cell infiltration. CIBERSORT is an analysis tool that uses gene expression data to estimate the abundance of member cell types in a mixed cell population (15). The LM22 gene file provided by CIBERSORT was used to define and infer the relative proportions of 22 types of immune-infiltrating cells in both disease and normal gene expression data (16). The default signature matrix of 100 permutations was used in the algorithm for this study (17). To ensure confidence in the results, CIRBERSORT uses Monte Carlo sampling to derive the deconvolution *p*-value for each sample, and, in this study, only data with *p*-values <0.05 were retained. The results were then visualized using the ggplot2 package in R, before performing a correlation analysis between the 22 immune cells and key genes using Spearman’s rank correlation test. Both MetS and DN are related to metabolism, and the set of metabolic signatures in the Molecular Signatures Database was used as a reference for gene set enrichment analysis, where *p* < 0.05 and a false discovery rate *q* < 0.05 were considered to indicate significant enrichment (18).

### 2.7. scRNA-seq data clustering dimension reduction

The merged data were first normalized using log normalization. Simultaneously, all genes were scaled using the ScaleData function, while the RunPCA function was used to reduce the principal component analysis (PCA) dimension for the first 2000 highly variable genes screened above. Dim = 20 was chosen before clustering the cells through the “FindNeighbors” and “FindClusters” functions (resolution = 0.8; Supplementary Figure S3) to find the cell clusters (19, 20). The top 50 principal components were then selected to further reduce dimensionality using the Uniform Manifold Approximation and Projection (UMAP) method. UMAP is a method of data dimensionality reduction, which assumes that the available data samples are uniformly distributed in the topological space and that these limited data samples can be approximated and mapped to a low-dimensional space. The “FindAllMarkers” function was subsequently used to screen the marker genes of 22 subgroups with |logFC| = 0.5 and min.pct = 0.35 (21). Finally, a corrected *p* < 0.05 was used to screen the marker genes.

### 2.8. Cell-type identification by estimating relative subsets of oxidative phosphorylation

OXPHOS is an electron transfer chain driven by substrate oxidation coupled to ATP synthesis through an electrochemical transmembrane gradient. Hallmark_oxidative_phosphorylation is a pathway based on the input matrix of gene expression file that is used to accurately estimate metabolism in tissues. This approach was used to compare metabolic differences between different cells in different groups. Spearman correlation analysis was performed to explore the relationship between metabolism and various cells in the kidneys of DN patients. The ggplot2 software package was then used to visualize the differences and results of related analyses (14, 22–26).

### 2.9. Statistical analysis

R version 4.1.3 was used for statistical analysis, while Student’s *t*-test was performed to assess significant differences among distinct groups. In addition, the glmnet R package was used for the LASSO and Cox regression analyses. *p*-values <0.05 indicated statistical significance (*p < 0.05; **p < 0.01; ***p < 0.001; ****p < 0.0001).

### 2.10. Ethics approval and informed consent

GEO is a public database, and ethical approval was obtained for the studies that collected the various data in the database. Users can freely download relevant data for use in research and publication of relevant articles. Our study was based on open-source data; therefore, there are no ethical issues or other conflicts of interest.

## 3. Results

### 3.1. Co-expression modules in DN and MetS

**Figure 1:**
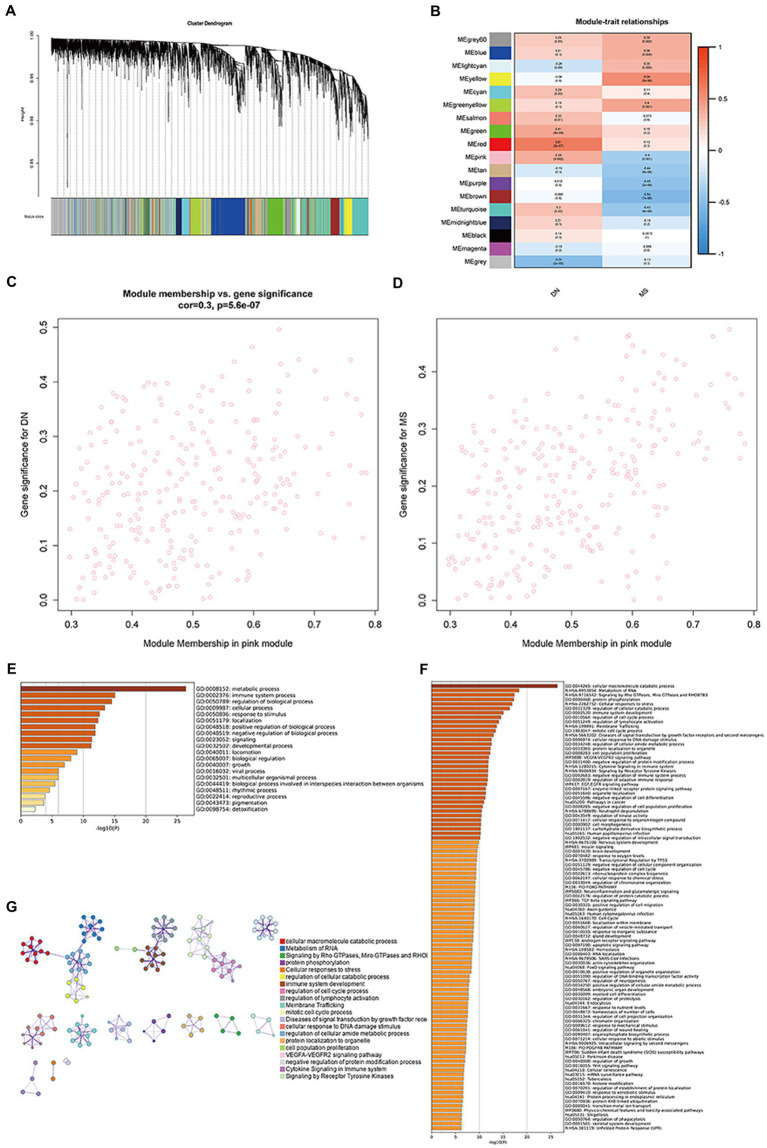
Co-expression modules in DN and MS. **(A)** Clustered dendrogram of genes. Each branch in the figure represents a gene, while each color below represents a co-expression module. **(B)** Heatmap of module-feature relationships. The pink module was significantly associated with DN. **(C)** Distribution of mean gene significance in modules associated with DN (*p* = 5.6e−07). **(D)** Distribution of mean gene significance in modules associated with MetS (*p* = 7.5e−17). **(E)** The top-level Gene Ontology biological processes. **(F)** Up to 100 enriched clusters. **(G)** Network of enriched terms: colored by cluster ID, where nodes that share the same cluster ID are typically close to each other.

Three datasets (GSE30529, GSE98895, and GSE99340) were integrated here, before removing batch effects. A soft threshold of *β* = 4 was chosen for consistency with the scale-free network. A total of 18 modules were identified in GSE30529 and GSE98895 using WGCNA, with each color representing a different module. A module-trait heatmap was also constructed according to the Spearman correlation coefficient to evaluate the association between each module and disease (Figures 1A,B). In the heatmap, the pink module showed the strongest association with DN (cor = 0.3, *p* = 5.6e-07; Figure 1C) and MetS (cor = 0.48, *p* = 7.5e−17; Figure 1D), which included 268 genes (Supplementary material 2). Enriched clusters up to 100 showed that the functionally enriched WGCNA pink and turquoise modules were primarily involved in the following processes: metabolic, immune system, and cellular processes, as well as the regulation of biological processes and response to stimuli. The molecular pathway was related to cellular macromolecule catabolic process, RNA metabolism, signaling by Rho and mitochondrial Rho GTPases and RHOBTB3, and protein phosphorylation (Figures 1E,F). Figure 1G further validates the correlation of comorbid WGCNA modules with metabolism and immunity.

### 3.2. Common gene signatures in DN and MetS

**Figure 2:**
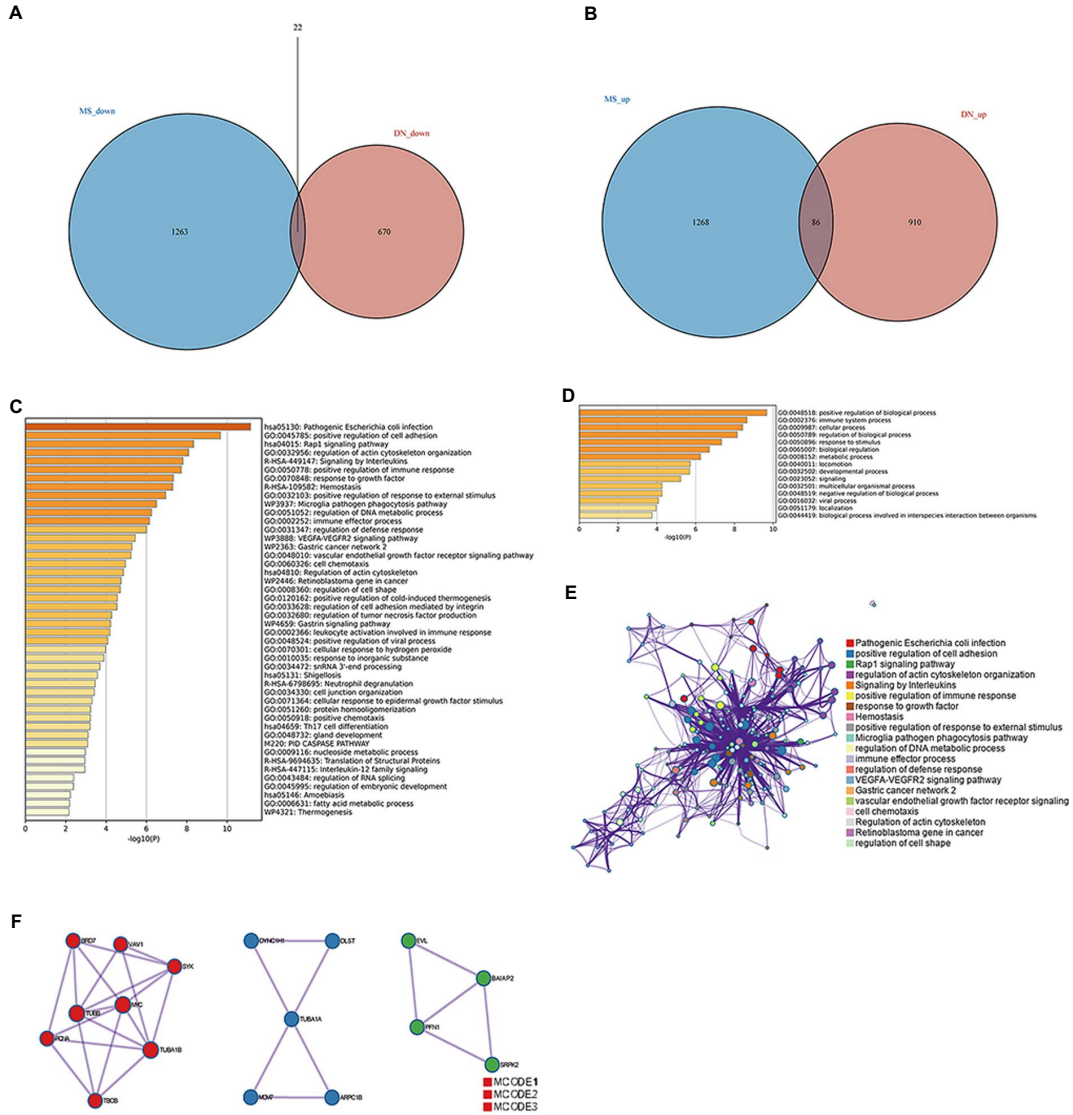
Common gene signatures in DN and MS. **(A)** Sixty-five upregulated genes related to DN and MS. **(B)** Thirty-four downregulated genes related to DN and MS. **(C)** The top-level Gene Ontology biological processes. **(D)** Up to 100 enriched clusters. **(E)** Network of enriched terms: Colored by cluster ID, where nodes that share the same cluster ID are typically close to each other. **(F)** Protein–protein interaction network and MCODE components identified in gene lists.

Up-and-down regulated DEGs were obtained for DN and MetS by setting the cut-off values at an adjusted *p*-value of 0.05 and |log_2_FC| > 1 (27). Ultimately, 86 upregulated and 22 downregulated genes associated with DN and MetS were discovered using the intersection of the differential genes of the two disorders (Figures 2A,B; Supplementary materials 3, 4). These genes were subjected to functional enrichment and module analyses using Metascape. Based on enrichment clusters up to 100, the common gene functions of DN and MetS were primarily enriched as follows: immune system function, cellular function, and regulation of biological processes (Figures 2C–E). The common DN and MetS related to metabolism and immunity were further investigated using MCODE, with three core modules subsequently obtained: module 1 included 8 genes: *SYK*, *VAV1*, *BRD7*, *TUBB*, *MYC*, *TUBA1B*, *PCNA*, and *TBCB*; module 2 included 5 genes: *DYNC1H1*, *DLST*, *TUBA1A*, *MCM7*, and *ARPC1B*; and module 3 included 4 genes: *EVL BAIAP2*, *SRPK2*, and *PFN* (Figure 2F).

### 3.3. Screening for common biological markers of DN and MetS

**Figure 3:**
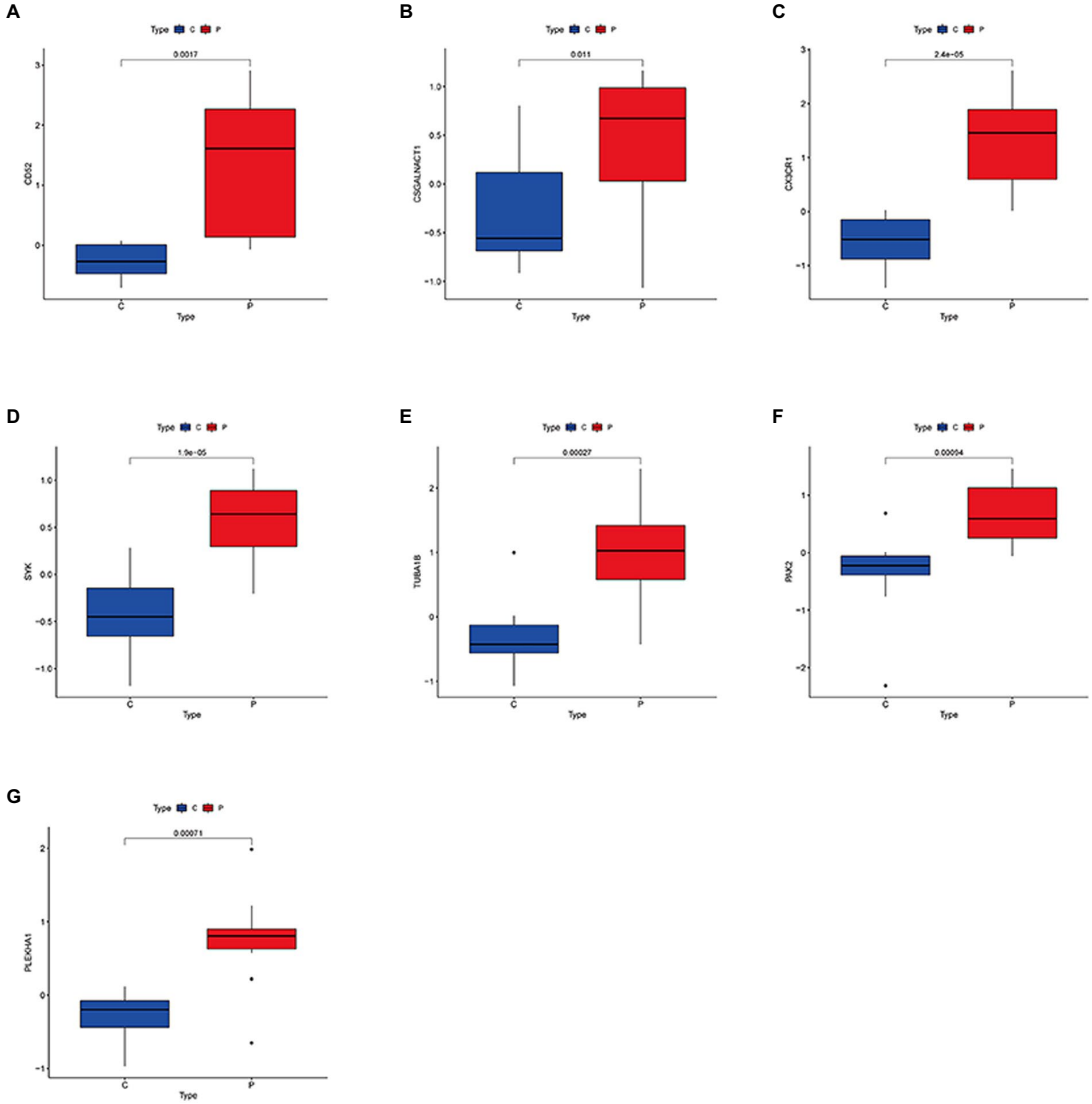
Expression levels of common biological markers in DN and MS. **(A–G)** *CD52*; *CSGALNACT1*; *CX3CR1*; *SYK*; *TUBA1B*; *PAK2*; *PLEKHA1.*

To further identify potential biomarkers for DN and MetS from the 108 target genes, Cox regression analysis of the LASSO data was used to reduce the number of candidate genes. The change in trajectory of each gene is shown in Supplementary Figure S4. Nine potential biological markers were obtained by LASSO regression, while seven potential biomarkers were obtained using the DN external validation dataset GSE99340. We also found that in GSE30529, seven genes were highly upregulated in DN samples (Figures 3A–G). As shown in Supplementary Figure S5, receiver operating characteristic (ROC) curves of the seven potential biological markers were well adapted in both the training and validation datasets (28). The Coef index for the seven identified genes is presented in Supplementary material 5. Ultimately, the seven genes were considered potential biomarkers for DN and MetS.

### 3.4. Building risk prediction models using XGBoost

**Figure 4:**
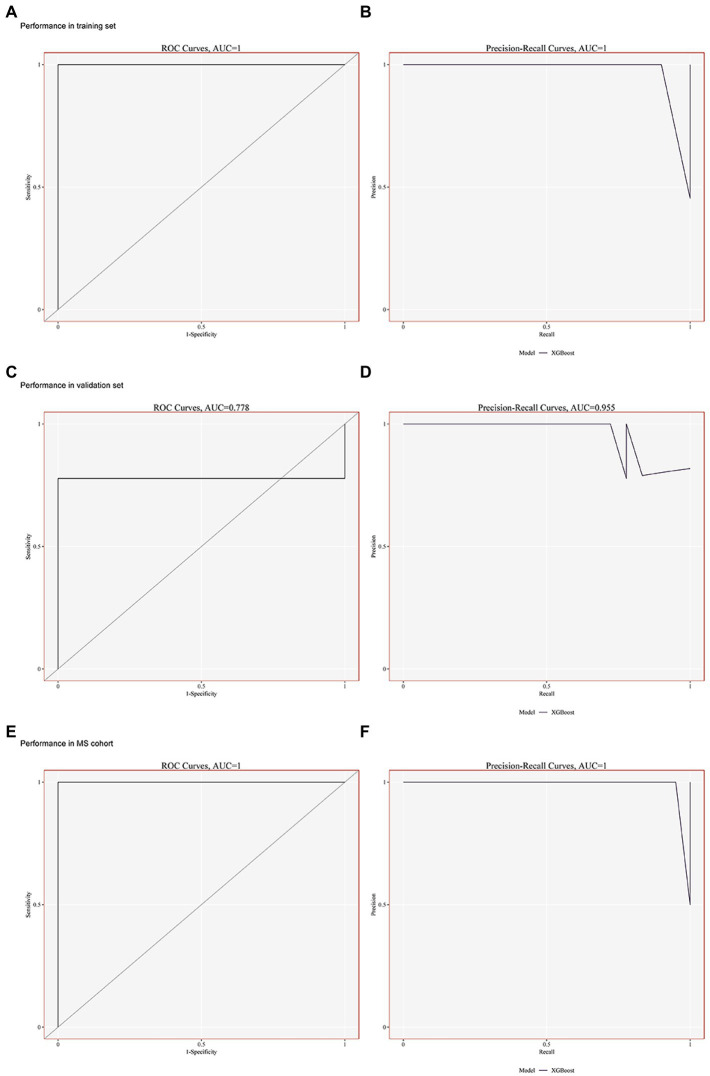
Building risk prediction models by XGBoost. (**A–B**) Performance of the ROC and PR curves in GSE30529 (training set). (**C–D**) Performance of the ROC and PR curves in GSE99340 (validation set). (**E–F**) Performance of ROC and PR curves in GSE98895 (MS cohort).

The XGBoost method was used to construct a lightweight model of the seven potential biological markers. Both the areas under the ROC curve for the DN training set GSE30529 and the precision-recall (PR) curve were 1 (Figures 4A,B). Furthermore, the area under the ROC curve in the validation set GSE99340 was 0.778, while that under the PR curve was 0.955 (Figures 4C,D). Simultaneously, XGBoost curve fitting was performed on the GSE988985 MetS dataset, yielding solid curve-fitting results (ROC = 0.995, PR = 0.995; Figures 4E,F). These results suggest that the seven potential biological markers exhibited favorable diagnostic efficacy for both MetS and DN.

### 3.5. Immune cell infiltration and metabolic pathway analysis

There was a difference in the composition of immune cells. Tumor microenvironment (TME) analysis results indicated the presence of immune infiltration in DN GSE30529, primarily memory B cells (MBCs), CD8^+^ T cells, γδ T cells, resting memory CD4^+^ T cells, and M_0_ macrophages (Supplementary Figure S6A). TME was grouped according to DN and control groups, with the results showing immune infiltration in DN GSE30529, mainly natural killer (NK) cells, monocytes, M_1_ macrophages, dendritic cells, and neutrophils (Supplementary Figure S6B). The immune-infiltrating cells in DN GSE30529 were predominantly macrophages, T cells, and MBCs (Supplementary Figures S6C,D). There was also a correlation between immune-infiltrating cells, with the results showing that γδ T cells and M_1_ macrophages were both highly correlated (cor = 0.85; Supplementary Figure S6E). The seven genes screened above were analyzed for their correlation with immune cells in the GSE30529 DN dataset, with the results showing that *SYK* highly correlated with γδ T cells and NK cells. *PHEKHA1* was also highly correlated with macrophages and resting memory CD4^+^ T cells (Supplementary Figure S6F). Furthermore, nine metabolic pathways were analyzed in the GSE30529 DN dataset. The upregulation of *SYK*, *CD52*, *CX3CR1*, *TUBA1B*, and *CSGALNACT1* led to the most noticeable metabolic abnormalities, which were reflected primarily in heme metabolism, bile acid metabolism, adipogenesis, OXPHOS, xenobiotic metabolism, and fatty acid metabolism (Supplementary Figure S7G). TME analysis also indicated the presence of immune infiltration in the GSE98895 MetS dataset, mainly monocytes, naïve CD4^+^ T cells, and CD8^+^ T cells (Supplementary Figure S7A). TME was grouped according to MetS and control groups, and the results showed immune infiltration in the GSE98895 MetS dataset, primarily monocytes, CD8^+^ T cells, and naïve CD4^+^ T cells (Supplementary Figure S7B). Monocytes and T cells constituted the major proportion of immune-infiltrating cells in the GSE98895 MetS dataset (Supplementary Figures S7C,D). GSE98895 immune-infiltrating cells were correlated, and the results showed that M_2_ macrophages and dendritic cells were highly correlated (cor = 0.84; Supplementary Figure S7E). In the GSE98895 MetS dataset, *CX3CR1* was associated with immune infiltration, with elevated *CX3CR1* levels leading to NK cell and monocyte activation. Furthermore, elevated expression levels of *PAK2* activated monocytes (Supplementary Figure S7F). *PLEKHA1*, *TUBA1B*, *CX3CR1*, *PAK2*, and *CD52* were the most highly correlated genes in the GSE98895 MetS dataset. *TUBA1B* expression was also elevated, resulting in OXPHOS, glycolysis, adipogenesis, xenobiotic metabolism, and fatty acid metabolism (Supplementary Figure S7G). Furthermore, *CX3CR1* expression was elevated, leading to increased OXPHOS, glycolysis, bile acid metabolism, xenobiotic metabolism, and fatty acid metabolism (Supplementary Figure S4G). *PAK2* expression was also elevated, leading to increased OXPHOS, xenobiotic metabolism, and fatty acid metabolism; moreover, *CD52* expression was elevated, leading to increased OXPHOS (Supplementary Figure S7G). Finally, *TUBA1B* expression was elevated, which led to increased OXPHOS, glycolysis, adipogenesis, xenobiotic metabolism, and fatty acid metabolism (Supplementary Figure S7G).

### 3.6. Definition of clusters and dimensionality reduction for visual representation of cells

**Figure 5:**
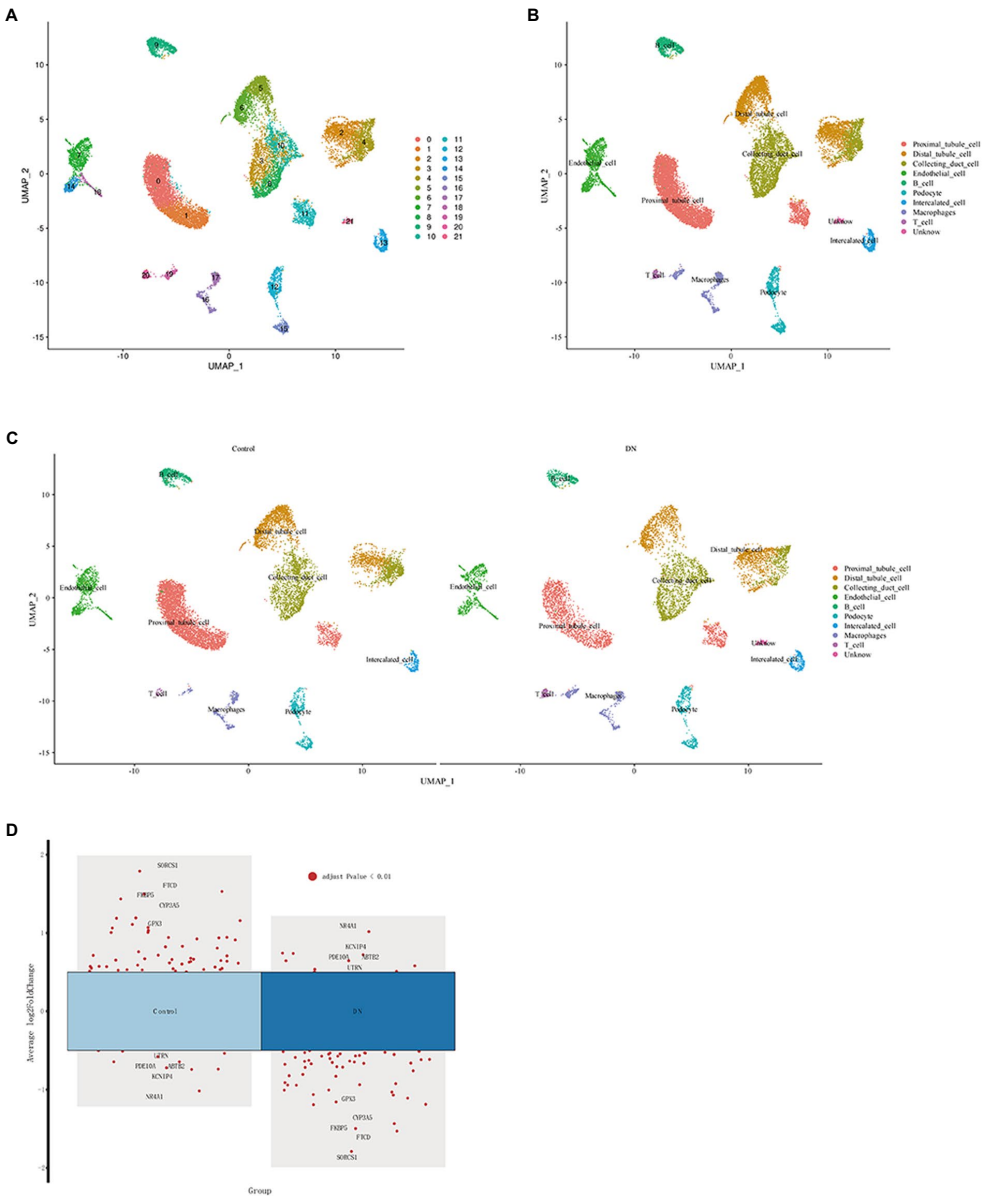
Definition of clusters and dimensionality reduction for visual representation of the cells. **(A)** UMAP of single cells from DN samples and normal samples before comments. **(B)** UMAP of single cells from DN samples and normal samples after the comment. **(C)** UMAP of single cells from DN samples and normal samples between DN and the control. **(D)** Single-cell analysis of differential genes in DN and control.

The “ScaleData” function was implemented to scale all genes extracted from the scRNA-seq dataset GSE131882 and PCA dimensionality reduction was performed to identify anchor points. A total of 21 clusters were identified (Figure 5A; Supplementary material 6). These identified clusters were then labeled as various cell types using the top five differential marker genes. Cell marker genes were downloaded from PanglaoDB^2^ and the Human Protein Atlas.^3^ Ultimately, 11 cell types were defined (Figure 5B), and after grouping into DN and blank controls, a single-cell analysis was performed (Figure 5C). The five differential genes with the most prominent contributions were screened, as shown in Figure 5D.

### 3.7. Common biological markers for single-cell analysis

**Figure 6:**
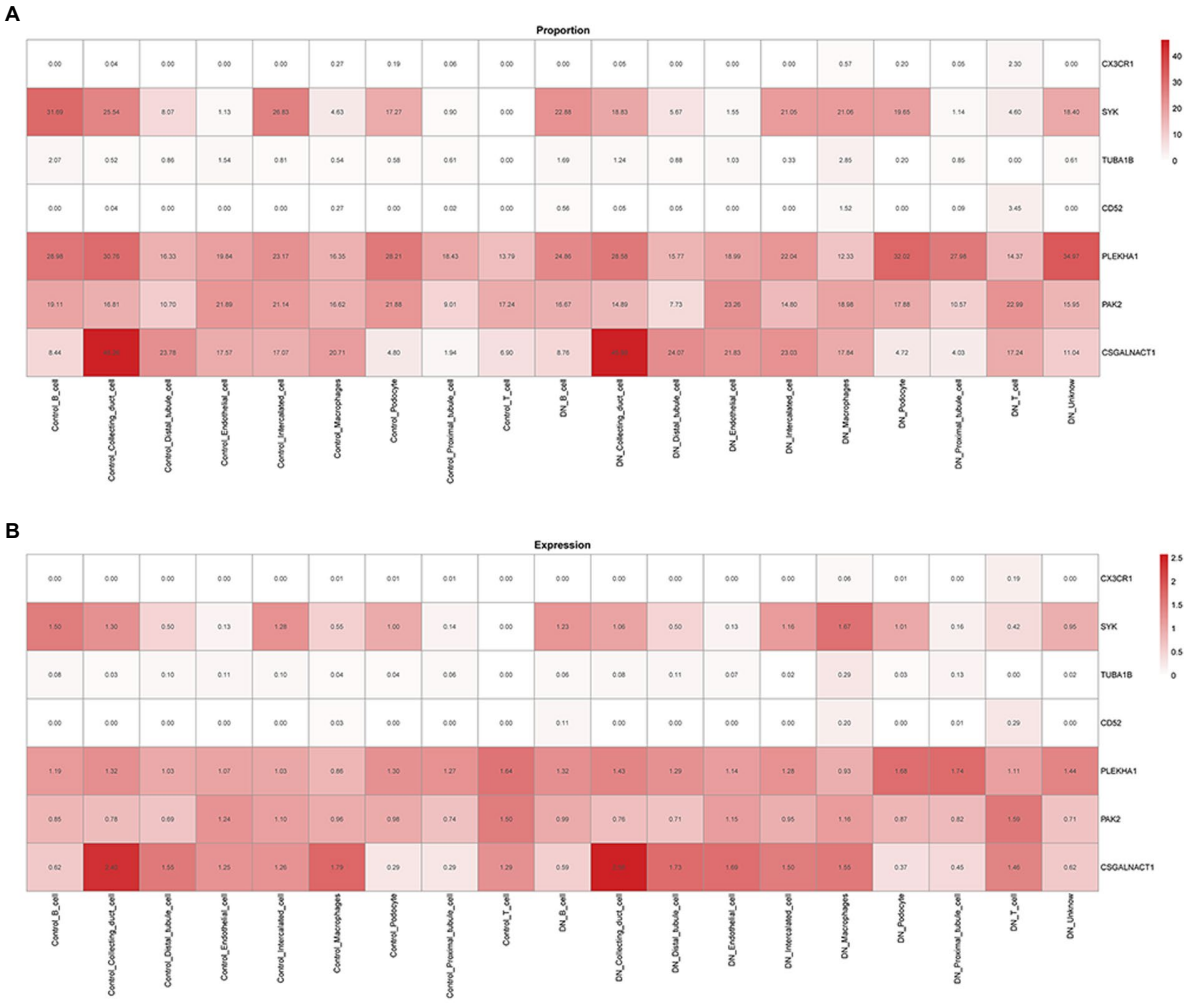
Relationship between biological markers. **(A)** The relationship between PLEKHA1 and CSGALNACT1. **(B)** The relationship between PLEKHA1 and PAK2.

The relationship between potential DN genes and single cells of DN after screening by proportional values subsequently showed that *CSGALNACT1*, *PAK2*, *PLEKHA1*, and *SYK* were all related to DN. Figure 6A shows the proportional relationship between the seven potential biological markers and single kidney cells. *SYK, PLEKHA1, PKA2,* and *CSGALNACT1* were found to have the highest associations with DN OXPHOS. Figure 6B shows the expression relationship between the seven potential biomarkers and individual kidney cells, demonstrating that the above four genes were the most relevant for OXPHOS in DN.

### 3.8. Association between *PLEKHA1* and single-cell OXPHOS in DN

**Figure 7:**
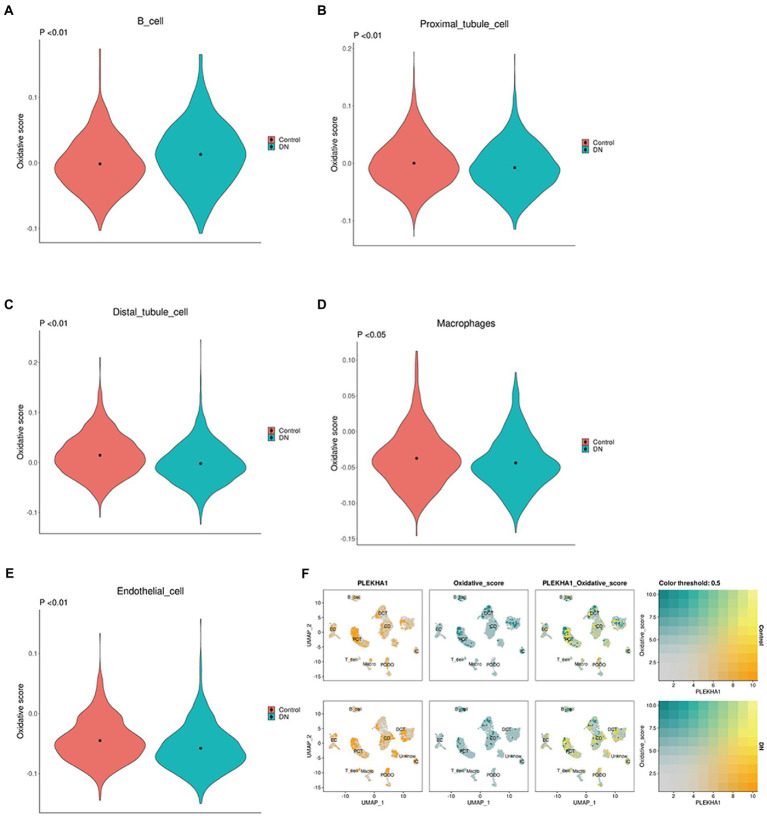
Association between the PLEKHA1 gene and single-cell oxidative phosphorylation. **(A–E)** Violin plot of potential biological markers and single-cell oxidative stress in DN: B cell. Proximal tubular cell. Distal tubular cell. Macrophages. Endothelial cell. **(F)** The relationship between PLEKHA1 and single-cell oxidative stress in DN was demonstrated by UMAP diagrams.

The relationship between upregulated *PLEKHA1* and the single-cell OXPHOS score for DN was presented in a violin plot; the results in Figures 7A–E show that upregulated *PLEKHA1* induced OXPHOS in B cells, proximal tubular cells, distal tubular cells, macrophages, and endothelial cells. Figure 7F also shows that the upregulation of PLEKHA1 led to elevated levels of OXPHOS in DN. Furthermore, Supplementary material 7 presents single-cell OXPHOS fractions for various types of DN.

## 4. Discussion

Diabetes mellitus is the most common cause of chronic kidney disease worldwide and can lead to multiple complications, including end-stage renal disease, cardiovascular disease, infection, and ultimately death (29, 30). MetS is a common metabolic disorder arising from the increasing prevalence of obesity (1). In this study, differential genes associated with DN and MetS were found to be primarily enriched in immune system function, cellular function, and biological process regulation. One of the potential biological markers identified in this study, *PLEKHA1*, induces OXPHOS in B cells, proximal tubular cells, distal tubule cells, macrophages, and endothelial cells.

The induction of oxidative stress, inflammation, fibrosis, and apoptosis by the accumulation of metabolites is known as lipotoxicity (31). Recent evidence has indicated that both the quantity and quality of lipids are involved in renal damage associated with lipotoxicity by activating inflammation, oxidative stress, mitochondrial dysfunction, and cell death. The pathological effects of lipotoxicity have also been observed in renal cells and promote podocyte injury, tubular damage, mesangial proliferation, endothelial activation, and macrophage-derived foam cell formation (32). Metabolic abnormalities and OXPHOS are the predominant contributors to DN.

DN is also associated with elevated expression levels of OXPHOS-related genes and pathways (33). Mitochondria are the site of cellular respiration and generate energy as ATP through OXPHOS; therefore, mitochondrial dysfunction has been implicated in DN. Mitochondria are also an important cellular source of reactive oxygen species (ROS) through the OXPHOS pathway (34, 35). Oxidative stress plays an important role in diabetic vascular complications (36), while hyperglycemia induces intracellular ROS production in interstitial cells and diabetic kidneys (37, 38). Based on indirect evidence, it has been hypothesized that increased oxidative stress contributes to the development of DN. *Smad4* promotes DN by regulating glycolysis and OXPHOS (39). Furthermore, OXPHOS, electron transport system complex III, the citric acid cycle, propionate metabolism, and transcription factors are all key contributors to metabolic abnormalities in DN (33). The paradigm that high glucose drives the overproduction of superoxide in mitochondria, as a unifying theory to explain end-organ damage in complications resulting from diabetes, has been upheld for more than a decade (40).

Considering its widespread prevalence and massive toll on health and finances, the diagnosis and management of DN are of great clinical and social relevance. Correlation and subgroup analyses of pivotal genes associated with the clinical features of DN showed that *ALB*, *ANXA1*, *APOH*, *C3*, *CCL19*, *COL1A2*, *COL3A1*, *COL4A1*, *COL6A3*, *CXCL6*, *DCN*, *EGF*, *HRG*, *KNG1*, *LUM*, *SERPINA3*, *SPARC*, *SRGN*, and *TIMP1* may all be involved in diabetic renal tubular interstitial injury (41). A urine transcriptome test also showed that urinary sediment *CCL5* and *CXCL1* mRNAs were upregulated in patients with DN, while being associated with a decline in renal function and the degree of renal interstitial fibrosis. Therefore, urinary sediment *CCL5* mRNA could be a potential prognostic biomarker of DN (42). Transcriptome analysis of osteoporosis in DN patients treated with traditional Chinese medicine showed that miR-574 may play an important role in DN-related osteoporosis, with the therapeutic effects of kaempferol and quercetin on Leri–Weill dyschondrosteosis in DN-related osteoporosis potentially mediated by miR-574 by targeting *MAPK1* (43).

In this study, we first identified potential biological gene markers of MetS and DN before further analyzing the metabolic and immune pathways involved in these conditions. The role of *PLEKHA1* in OXPHOS, which is involved in DN, was investigated *via* single-cell analysis, and *PLEKHA1* was identified as a potential biological marker of DN worthy of further study.

SGLT2 inhibitors have potential as an antifibrotic therapeutic intervention by regulating inflammation, oxidative stress, mitochondrial function, and autophagy. GLP-1 agonists reduce inflammation and oxidative stress, which can damage the kidneys in people with diabetes. CCR2/5 inhibitors block the chemokine receptors CCR2/CCR5, reducing immune cell infiltration, cytokine production, and slowing DN progression (44, 45). However, more research is needed to verify their safety and efficacy in the treatment of fibrotic diseases (46). Our research highlighted PLEKHA1 as a key gene in the development of DN. Future studies should investigate the effects of drug treatment on single cells of patients with diabetes to validate PLEKHA1 as a therapeutic target and inform the development of targeted therapies.

Our study of the shared biological markers of DN and MetS is critical to understanding the comorbidities between these conditions. To increase our sample size, we used transcriptome datasets of DN from the GEO database and adjusted for batch effects. We incorporated both transcriptome and single-cell analyses, allowing us to draw a definite conclusion. However, our study is not without limitations. We could not collect clinical data, which limited our ability to conduct further analysis on clinical prognosis. Furthermore, our study focused solely on the genetic aspect and lacked a control treatment group. These limitations underscore the need for future research to focus on clinical applications and treatments to enhance the practical implications of our findings.

In conclusion, a functional enrichment analysis was performed based on the common patterns of differential gene expression between DN and MetS, and these differential genes were found to be related to immunity and metabolism. Further screening of potential biological markers resulted in the identification of seven well-fitting genes. Subsequently, the relationship between *PLEKHA1* and OXPHOS in DN was further investigated by single-cell analysis. This study shows that overexpression of PLEKHA1 in B cells, proximal tubular cells, distal tubular cells, macrophages, and endothelial cells caused oxidative stress in the kidneys. The role of *PLEKHA1* in OXPHOS, which is involved in DN, was investigated *via* single-cell analysis, and *PLEKHA1* was identified as a potential biological marker of DN worthy of further study.

## Data availability statement

Publicly available datasets were analyzed in this study. This data can be found here: GSE30529, GSE98895, GSE99340, and GSE131882 GEO Accession viewer https://www.ncbi.nlm.nih.gov/geo/query/acc.cgi?acc=GSE30529. GEO Accession viewer https://www.ncbi.nlm.nih.gov/geo/query/acc.cgi?acc=GSE98895. GEO Accession viewer https://www.ncbi.nlm.nih.gov/geo/query/acc.cgi?acc=GSE99340. GEO Accession viewer https://www.ncbi.nlm.nih.gov/geo/query/acc.cgi?acc=GSE131882.

## Ethics statement

Ethical review and approval was not required for the study on human participants in accordance with the local legislation and institutional requirements. Written informed consent for participation was not required for this study in accordance with the national legislation and the institutional requirements.

## Author contributions

CZ performed the study, collected the data, and wrote the paper. HL conceived and designed the study and revised the paper. SW curated the data and administered the project, and revised the paper. All authors contributed to the final manuscript and approved the submitted version.

## Conflict of interest

The authors declare that the research was conducted in the absence of any commercial or financial relationships that could be construed as a potential conflict of interest.

## Publisher’s note

All claims expressed in this article are solely those of the authors and do not necessarily represent those of their affiliated organizations, or those of the publisher, the editors and the reviewers. Any product that may be evaluated in this article, or claim that may be made by its manufacturer, is not guaranteed or endorsed by the publisher.

## Footnotes

^1^ http://metascape.org

^2^ https://panglaodb.se/index.html

^3^ https://www.proteinatlas.org/
