# Supplementary figures and images for: Common gene signatures and molecular mechanisms of diabetic nephropathy and metabolic syndrome
Source: Front Public Health. 2023 Mar 30;11:1150122. doi: 10.3389/fpubh.2023.1150122 (PMC10151256; doi:10.3389/fpubh.2023.1150122)

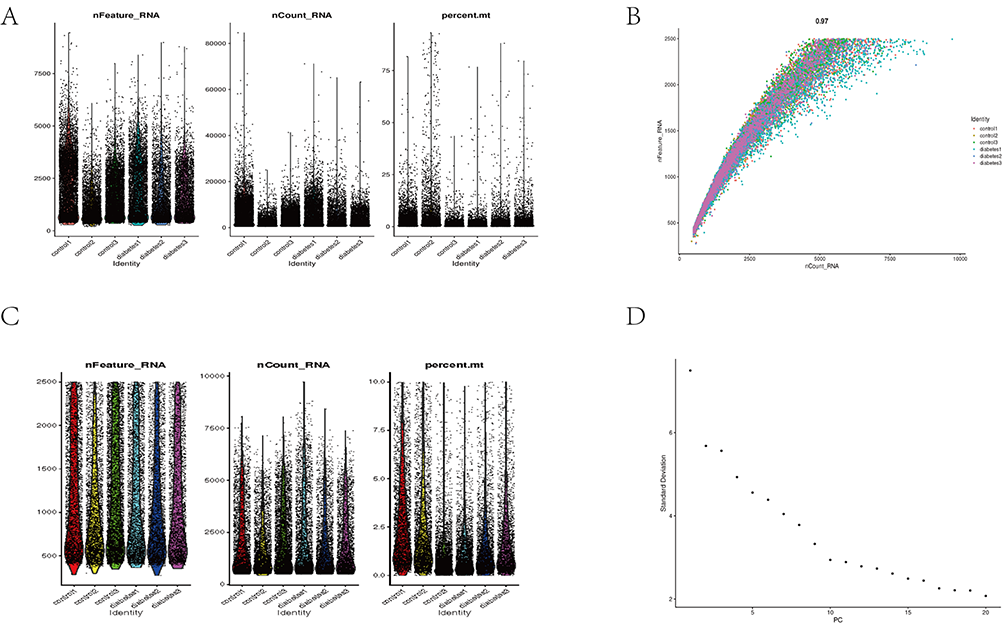

Supplement: Supplementary file 8 [file Image_1.TIF]

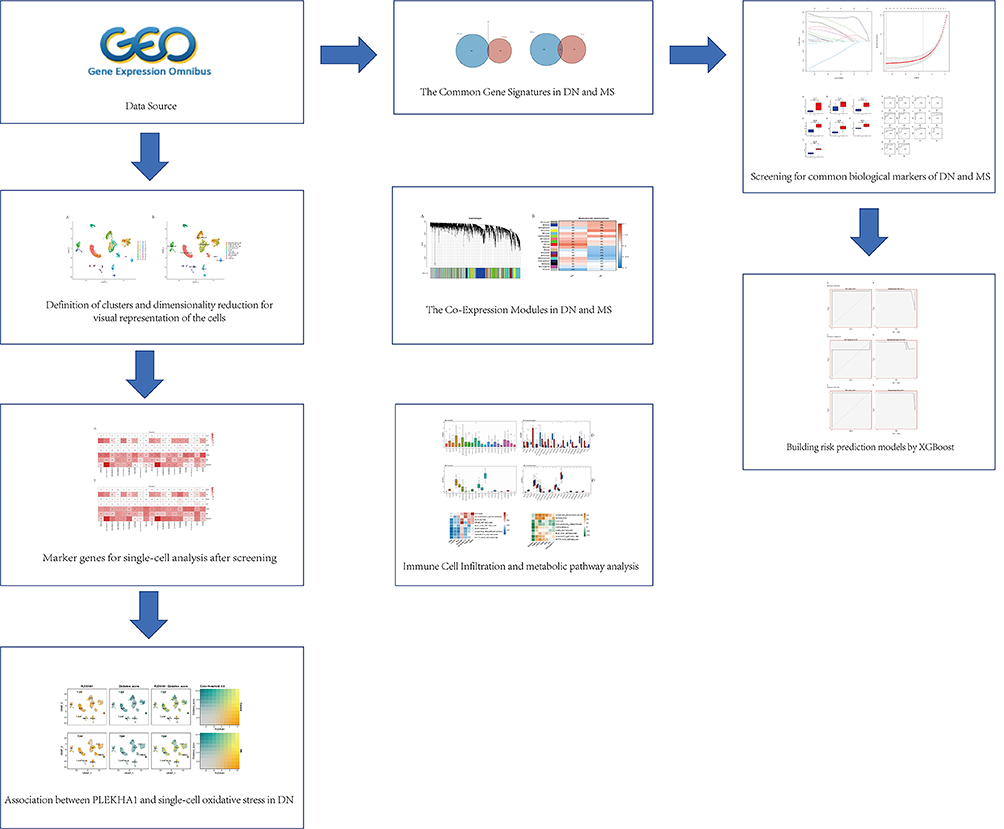

Supplement: Supplementary file 9 [file Image_2.TIF]

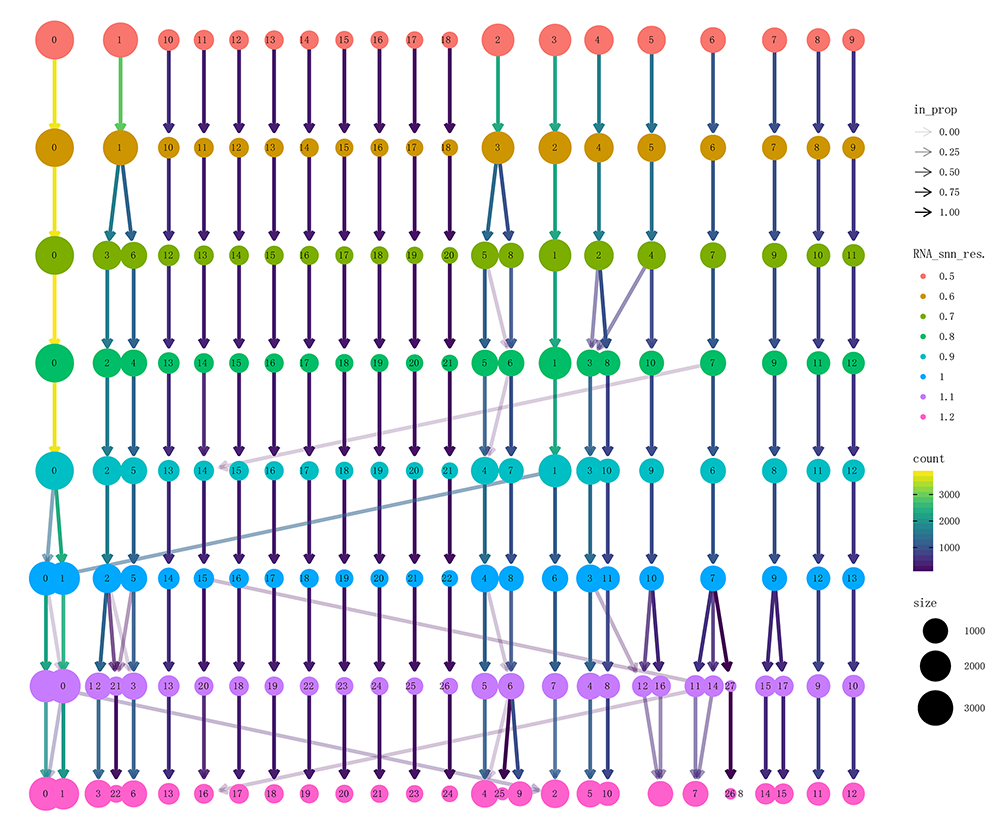

Supplement: Supplementary file 10 [file Image_3.TIF]

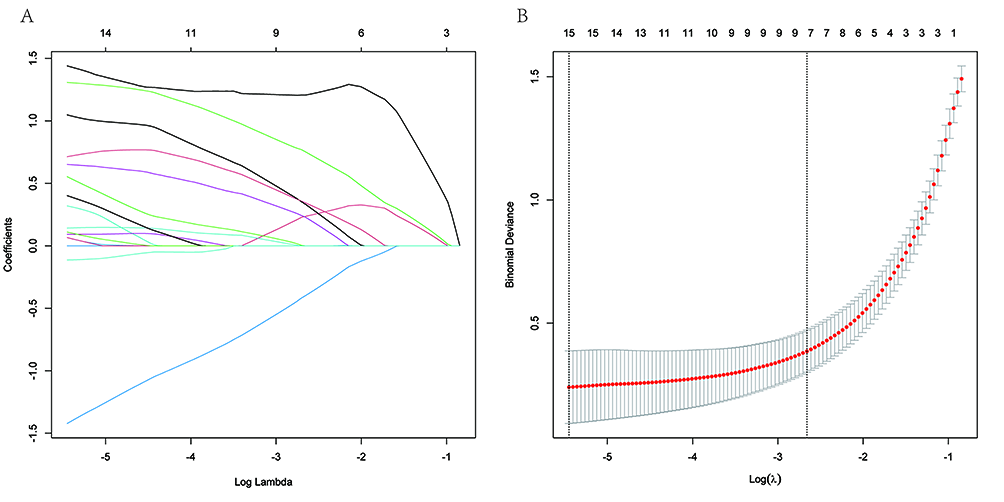

Supplement: Supplementary file 11 [file Image_4.TIF]

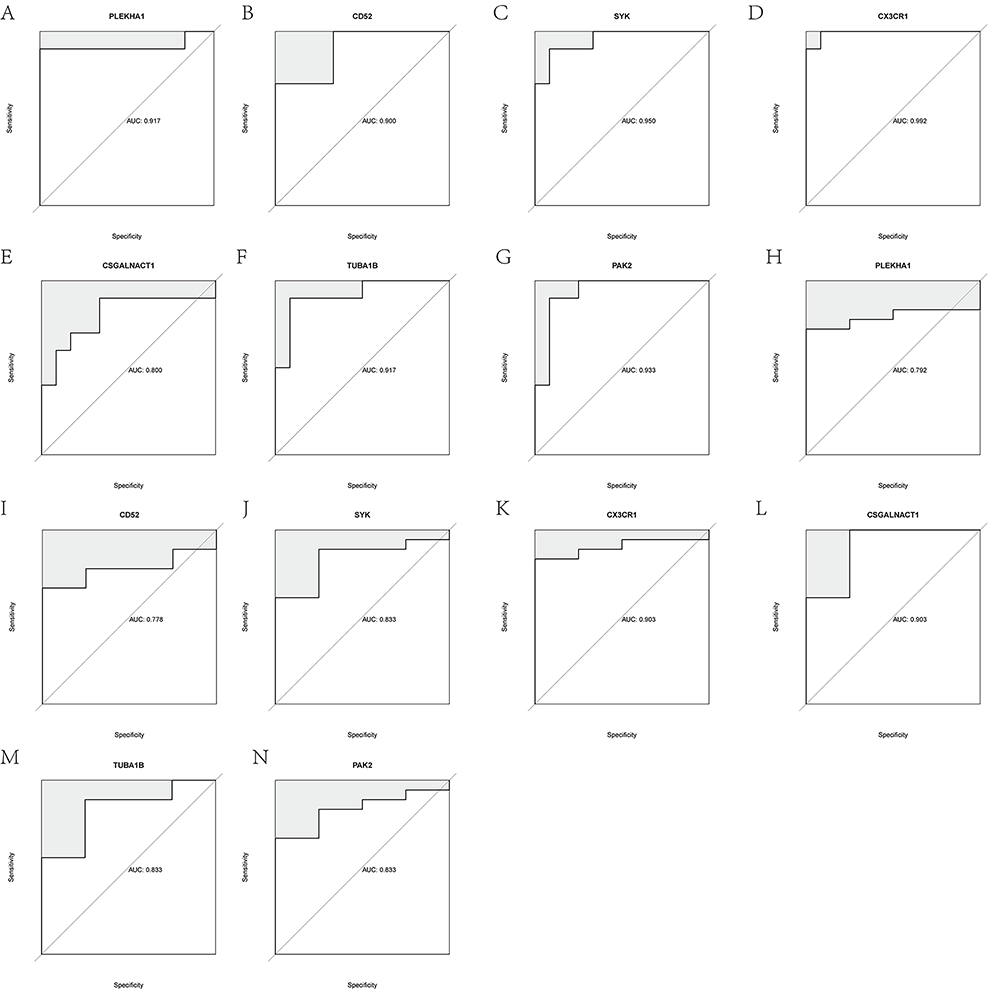

Supplement: Supplementary file 12 [file Image_5.TIF]

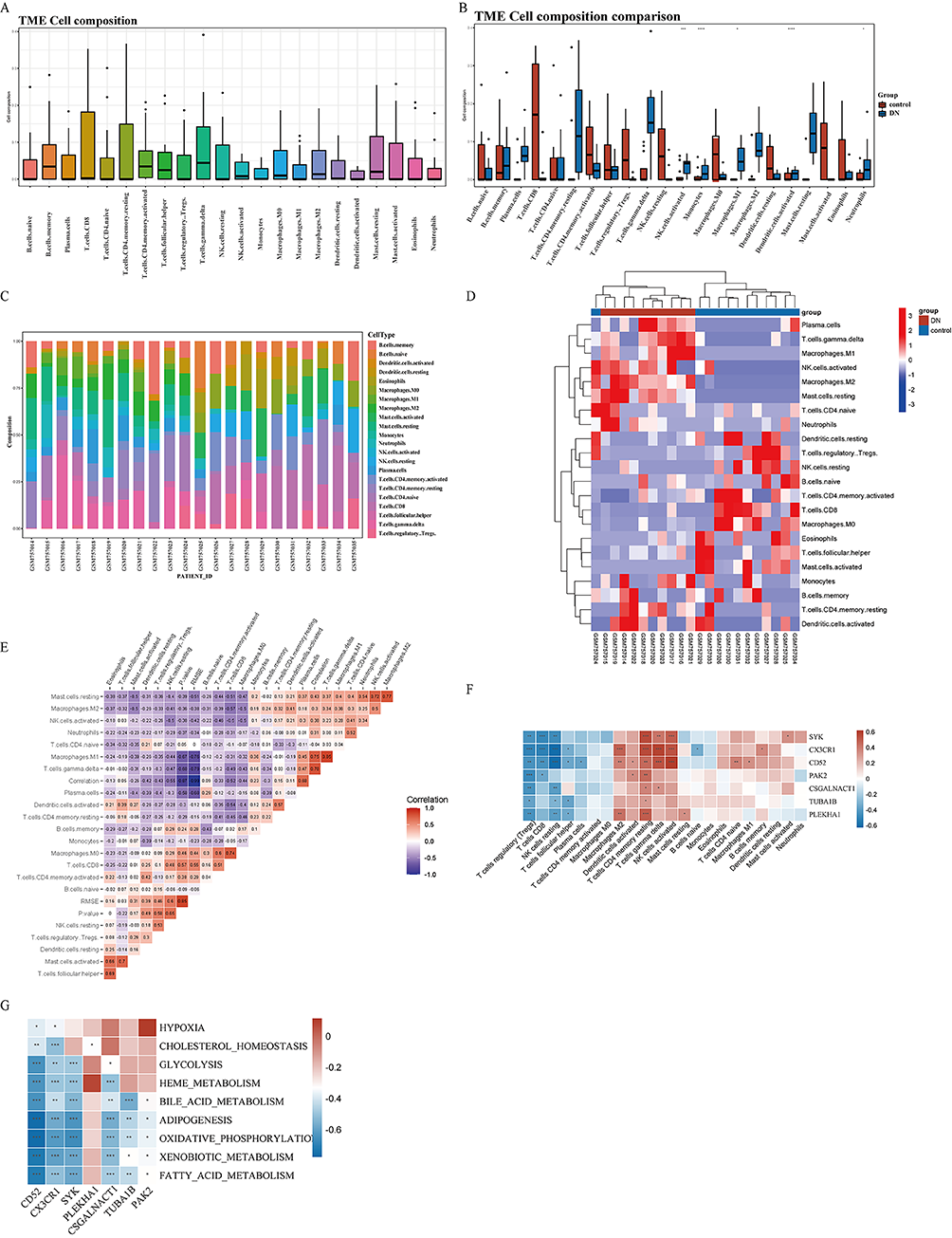

Supplement: Supplementary file 13 [file Image_6.TIF]

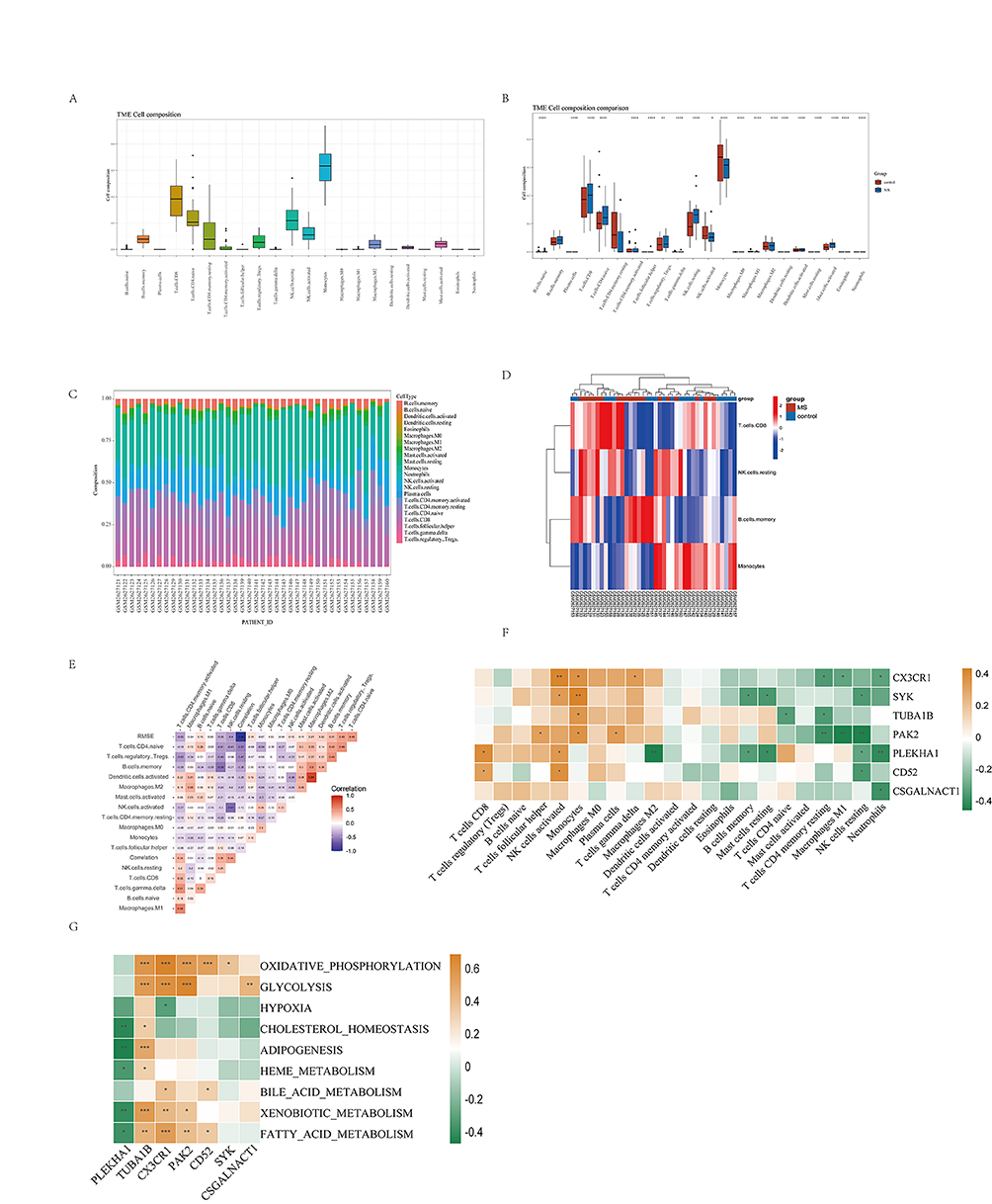

Supplement: Supplementary file 14 [file Image_7.TIF]
